# Integrating gonadal RNA-seq and small RNA-seq to analyze mRNA and miRNA changes in medaka sex differentiation

**DOI:** 10.1038/s41597-025-05129-y

**Published:** 2025-05-12

**Authors:** Xing Lin, Yuanli Zhao, Yifan Bai, Kaifeng Meng, Yuanyuan Chen, Meidi Hu, Fei Liu, Daji Luo

**Affiliations:** 1 State Key Laboratory of Breeding Biotechnology and Sustainable Aquaculture, Institute of Hydrobiology, Hubei Hongshan Laboratory, Chinese Academy of Sciences, Wuhan, 430072 China; 2 College of Advanced Agricultural Sciences, University of Chinese Academy of Sciences, Beijing, 100049 China; 3 College of Fisheries, Huazhong Agricultural University, Wuhan, 430070 China; 4 Fisheries College, Ocean University of China, Qingdao, 266001 China; 5 State Key Laboratory of Freshwater Ecology and Biotechnology, Institute of Hydrobiology, Chinese Academy of Sciences, Wuhan, 430072 China

**Keywords:** RNA sequencing, miRNAs

## Abstract

MicroRNAs are important post-transcriptional regulators, yet the molecular crosstalk between miRNAs and their target genes during sex differentiation remains poorly understood. Medaka (*Oryzias latipes*), the first fish in which the sex determination gene was identified, serves as an ideal model for studying this process. Here, we generated gonadal RNA-seq and small RNA-seq data from XY^*DMY*-^ females, wild-type females and males to explore this crosstalk. A total of twenty-seven RNA-seq datasets, comprising 188 Gb of raw reads, and twenty-seven small RNA-seq datasets, totaling 18 Gb of raw reads, were collected, covering 10, 30 and 120 days. After optimizing the mapping and normalizing, we conducted transcriptional and post-transcriptional dynamic analyses of differentially expressed genes and miRNAs between WT females and males, as well as between WT females and XY^*DMY-*^ females. Additionally, we integrated the RNA-seq and small RNA-seq data to construct comprehensive interaction networks and performed a detailed analysis of the temporal dynamics in gene and miRNA expression. These resources offer valuable insights into the transcriptional regulation of gonadal differentiation and development in vertebrates.

## Background & Summary

Fish play a pivotal transitional role in the evolutionary history of vertebrates, with their sex determination strategies covering nearly all observed mechanisms in vertebrates^1,2^. Medaka, which *DMY/dmrt1by* was discovered as the sex determination gene, serves as the optimal model for investigating the molecular mechanisms underlying sex determination and differentiation^3–5^. Over the last three decades, advancements in the genome project^6^, transcriptomics^7,8^, chromatin accessibility^9^, and gene editing^10,11^ have significantly advanced research on medaka. Recent studies have constructed XY^*DMY-*^ female medaka to investigate how *DMY* may function and influence transcription^12^. An increasing number of studies suggest that post-transcriptional regulation plays a crucial role in sex determination and differentiation^13,14^. However, its specific regulatory functions in vertebrates, including medaka, remain undefined.

Post-transcriptional regulation refers to the regulation of gene expression at the post-transcriptional level, including alternative splicing^15,16^, lncRNA^17^, m6A^18,19^, and miRNA^20^. Early studies reported that miR-430 accelerates the deadenylation of target mRNAs^21^ and modulates the number of primordial germ cells in zebrafish^22^. Subsequently, it was found that miR-202-5p, the most common mature form of miRNA in the ovaries of common carp^23^, medaka^24^ and zebrafish^25^, is essential for the development of follicles in juvenile females and for fertility in adult females^24^. These implied that miRNA serve as important post-transcriptional regulatory mechanisms during sex differentiation by facilitating mRNA degradation^26^. Consequently, although many studies focus on the dynamic expression of miRNAs^27^, research integrating mRNA and miRNAs to explore their interconnected regulatory mechanisms remains limited. Thus, we combined gonadal transcriptome and small RNA-seq data to provide a resource for further investigating the post-transcriptional mechanism involved in gonadal differentiation in medaka.

**Fig. 1:**
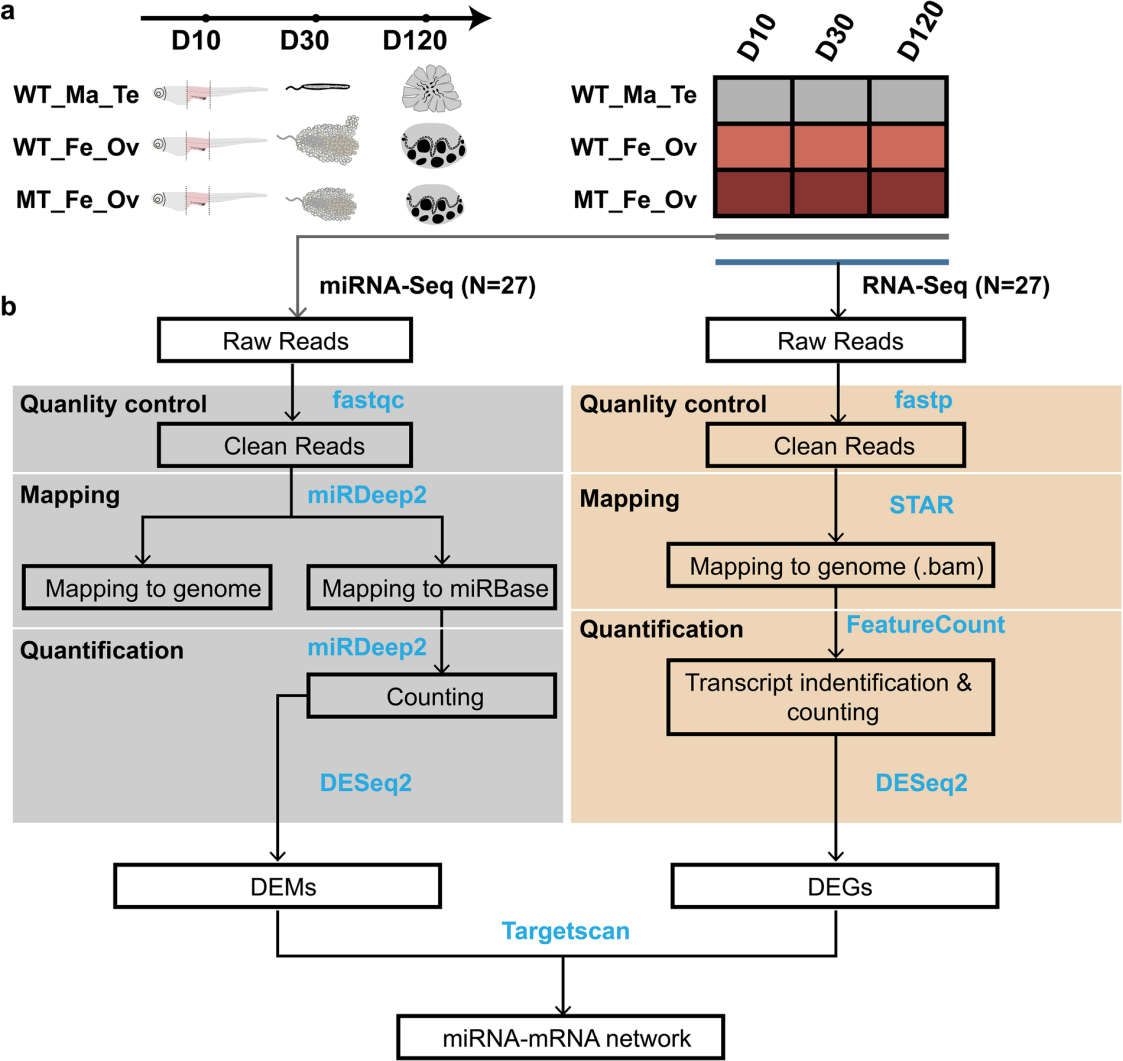
A schematic overview of the RNA-seq and small RNA-seq integration analysis. (**a**) Twenty-seven sets of RNA-seq and twenty-seven sets of small RNA-seq data covering three genotypes in medaka (WT_Ma_Te, WT_Fe_Ov, and MT_Fe_Ov) at three phases of gonadal differentiation (D10, D30, and D120) were collected. WT_Ma_Te: the testis in the male medaka of the wild type; WT_Fe_Ov: the ovary in the female medaka of the wild type; MT_Fe_Ov: the ovary in the female medaka of the mutant type. D10, D30, and D120 stand for 10, 30, and 120 days post hatch, respectively. (**b**) The analysis pipeline for small RNA-seq, RNA-seq, and mRNA-miRNA networks. Filtering out low-expression genes and miRNAs with read counts <1.

We previously established XY^*DMY-*^ female medaka to investigate the functional role of *DMY* in transcriptional regulation^12^. To further explore the molecular interactions between *DMY* gene, miRNAs and downstream genes networks, we performed transcriptional and post-transcriptional profiling at three stages: 10 days post-hatch (D10), 30 days post-hatch (D30), and 120 days post-hatch (D120) in XY^*DMY-*^ females (MT_Fe_Ov), WT females (WT_Fe_Ov) and males (WT_Ma_Te) (Fig. 1). A total of 54 RNA-seq and miRNA-seq datasets were generated, with standardized processing pipelines. By analyzing these datasets, we identified differentially expressed genes (DEGs), differentially expressed miRNAs (DEMs), and reconstructed dynamic miRNA-mRNA regulatory networks associated with *DMY* gene in medaka. This resource provides critical insights into the interplay between sex determination genes, mRNA dynamics, and miRNA-mediated post-transcriptional mechanisms, offering a foundational framework for studying vertebrate sex determination and gonadal development.

### Ethics Statement

The research animals receive optimal feeding and ministration under the supervision of a professional worker. The Institute of Hydrobiology, Chinese Academy of Sciences, approved all procedures and conducted them in accordance with the Guiding Principles for the Care and Use of Laboratory Animals.

## Methods

### Fish maintain and sample collection

XY^*DMY*-^ females medaka were generated using transcription activator-like effector nucleases (TALEN)^12^, with stable inheritance confirmed over ten generations. Medakas were maintained at 28.5 °C under a 14 hours light/10 hours dark cycle (lights on at 8:00 a.m.) at the Institute of Hydrobiology, Chinese Academy of Science.

Based on the medaka sex differentiation, three stages (D10, D30, and D120) were selected as sampling points, with WT_Ma_Te, WT_Fe_Ov, and MT_Fe_Ov as experimental groups. For D10 juveniles, gonadal primordia and adjacent tissues were collected, excluding the head and tail. The tail segment was used for genomic DNA extraction, enabling sex identification and genotyping (XX, XY, XY^*DMY*-^) through *DMY* gene amplification^12^. Tissues from three biological replicates were processed to construct RNA-seq and small RNA-seq libraries for downstream analysis.

### Preparation of cDNA library and Illumina sequencing

Total RNA was extracted using TRIzol reagent (Invitrogen, USA) according to established methods, and the detection of purity, quantity, and integrity of total RNA has been previously described^28^. Total RNA samples were separated into mRNA and small RNA and used for transcriptome sequencing and small RNA sequencing, respectively.

Utilizing Poly (dT) oligo-attached magnetic beads, the mRNA was extracted from the total RNA and enriched. cDNA libraries were then prepared using the TruSeq RNA Sample Preparation Kit (Illumina, USA). Finally, the cDNA libraries were sequenced using the Illumina Hiseq 2000^TM^ platform after PCR amplification.

The conventional methodology was followed in the construction of the small RNA libraries. To put it briefly, polyacrylamide gel electrophoresis was used to separate short-length RNAs, measuring 17 to 32 bp, from the total RNA. After the chosen segments were ligated to 5′ and 3′ RNA adapters, RT-PCR amplification was carried out. Finally, the PCR products were sequenced using the Illumina Hiseq 2000^TM^ platform.

### Read mapping and normalization of RNA-seq data

**Fig. 2:**
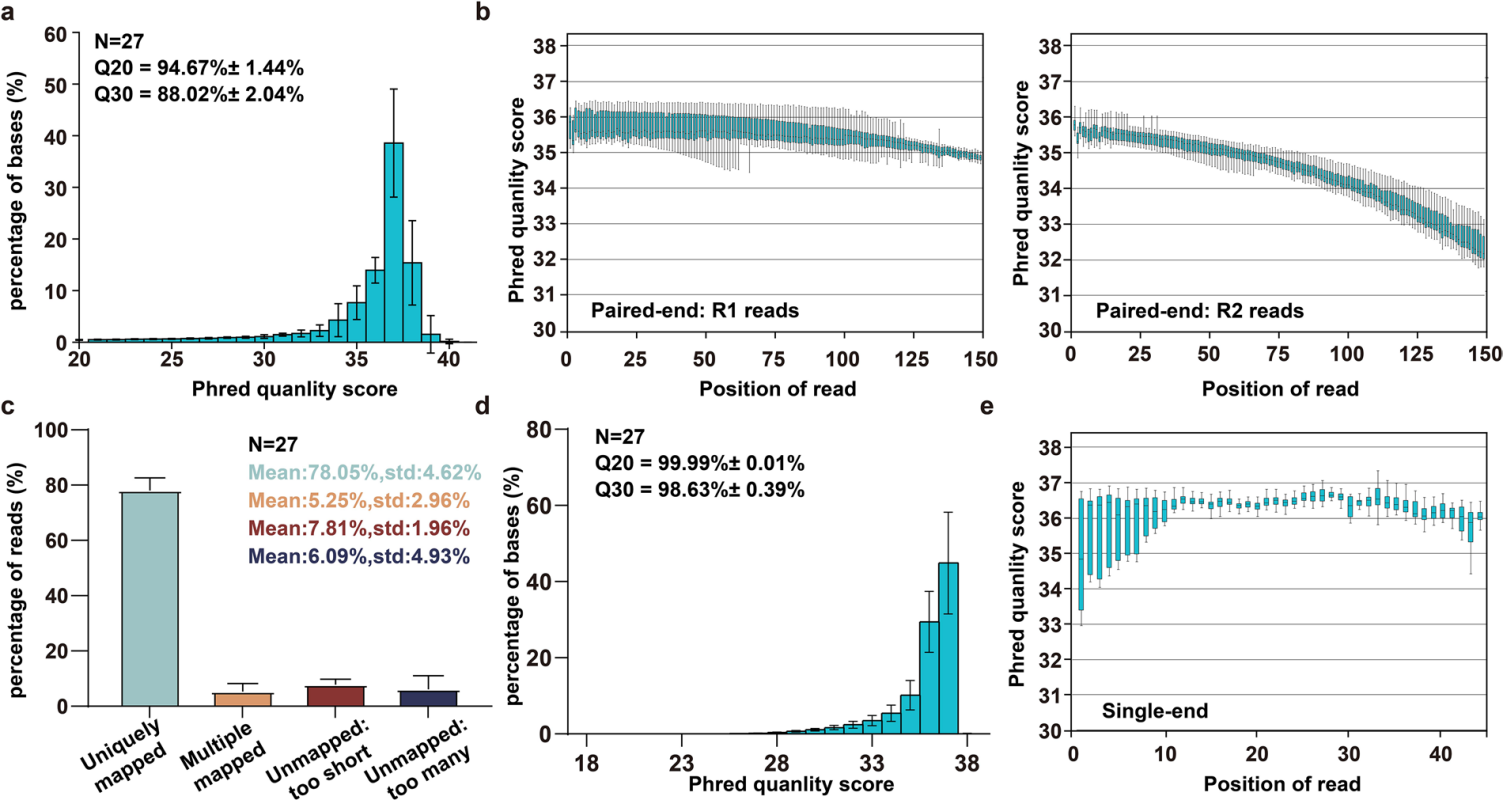
Summary of sequencing and genome alignment data. (**a**) The distribution of the mean quality of the RNA-seq reads. (**b**) The base quality of the paired-end R1 and R2. Data are presented as median ± minimum to maximum. (**c**) Summary of genome alignment for RNA-seq. Uniquely mapped means reads successfully mapped to one genomic location; Multiple mapped means reads successfully mapped to multiple genomic locations; Unmapped: too short means that failed to be mapped because the read was too short; Unmapped: too many means reads that failed to be mapped because of too many mismatches. (**d**) The distribution of the mean quality of the small RNA-seq reads. (**e**) The base quality of the singed end reads. Data are presented as median ± minimum to maximum.

To remove low-quality reads, RNA-seq data were processed with fastp v0.12.4^29^ using the default parameters (Fig. 2a, b). As the official pipeline tool of the ENCODE project, STAR^30^ has higher accuracy and faster operation efficiency. Therefore, this study used STAR (v2.7.5c) software to align RNA-seq reads with the Medaka genome (ASM223467v1) to improve the dependability (Fig. 2c). The end-to-end alignment was carried out applying the following settings: “--runMode alignReads --outSAMtype BAM SortedByCoordinate --alignEndsType EndToEnd --outFilterMultimapNmax 10 --readFilesCommand zcat--outFileNamePrefix --genomeDir --readFilesIn --quantMode TranscriptomeSAM GeneCounts”. Employing featureCounts (v2.0.1) command “-T threads -p -C -B -t exon -g gene -a gtf -o output bam” to aggregate the raw counts of mapped reads. Fragments per kilobase of exon model per million (FPKM) data were generated using the *fpkm* function in DESeq2^31^, and then the FPKM values were employed for principal component analysis (PCA) and cluster analysis, filtering out low-expression genes with read counts < 1.

Differentially expressed genes (DEGs) among WT_Fe_Ov, WT_Ma_Te, and MT_Fe_Ov at three phases (D10, D30, and D120) were identified by DESeq2^31^. In summary, the analysis was done for nine groups (WT_Fe_Ov vs. WT_Ma_Te, MT_Fe_Ov vs. WT_Ma_Te, and WT_Fe_Ov vs. MT_Fe_Ov at the D10; WT_Fe_Ov vs. WT_Ma_Te, MT_Fe_Ov vs. WT_Ma_Te, and WT_Fe_Ov vs. MT_Fe_Ov at the D30; WT_Fe_Ov vs. WT_Ma_Te, MT_Fe_Ov vs. WT_Ma_Te, and WT_Fe_Ov vs. MT_Fe_Ov at the D120). Genes were considered differentially expressed when the adjusted *P*-value was ≤ 0.01 and the absolute value of log_2_[foldchange] was ≥ 1. We used fuzzy clustering analysis to gain a deeper understanding of the expression patterns of three genotypes at three developmental stages. Genes were sorted according to their fuzzy clustering patterns using heatmap, and GO (Gene Ontology) enrichment was performed by ClusterProfiler v3.12.0^32^ to obtain each cluster’s function. The *P*-value ≤ 0.05 was used as the cutoff criterion.

### Read mapping and normalization of small RNA-seq data

Raw small RNA-seq reads were initially processed to yield high-quality reads with lengths longer than 17 nt. The library was then analyzed for quality using FastQC (www.bioinformatics.babraham.ac.uk/projects/fastqc/) to delete reads with Q values < 20 (Fig. 2d, e). Clean reads were mapped to the Medaka genome (ASM223467v1) using the mapper.pl module from miRDeep v2.0.1.2^33^ with the parameters: -c -j -l -m -p -s -t -o. miRNA quantification was performed using the quantifier.pl program (parameters:“-p hairpin.fa -m mature.fa -r -y”) to analyze known miRNAs from FishmiRNA database^34^. The reads per million mapped reads (RPM) values are calculated with the following formula: $R{PM}=\frac{c{ount}\times {10}^{6}}{{\rm{L}}{ibrarysize}}$, and then the RPM values were used to perform cluster and PCA analysis after non-expressed miRNAs were filtered out.

miRNA targets in medaka were predicted using the TargetScan package (v7.0)^35^. There are all 18,205 medaka 3′UTR sequences from Ensembl (Release 113) and curated 437 medaka miRNAs from the FishmiRNA database (v2.0). A novel computational workflow was developed to perform *de novo* prediction by modifying targetscan_70.pl (EasyTargetScan_0.2.py)^36^ to use medaka-specific parameters (Taxon ID: 8090). DESeq2^31^ was employed to identify differentially expressed miRNAs (DEMs) across nine pairwise comparisons among WT_Fe_Ov, WT_Ma_Te, and MT_Fe_Ov at three stages (D10, D30, and D120), following the same analytical pipeline used for DEGs.

### Transcriptional and post-transcriptional dynamic analysis of DEGs and DEMs

**Fig. 3:**
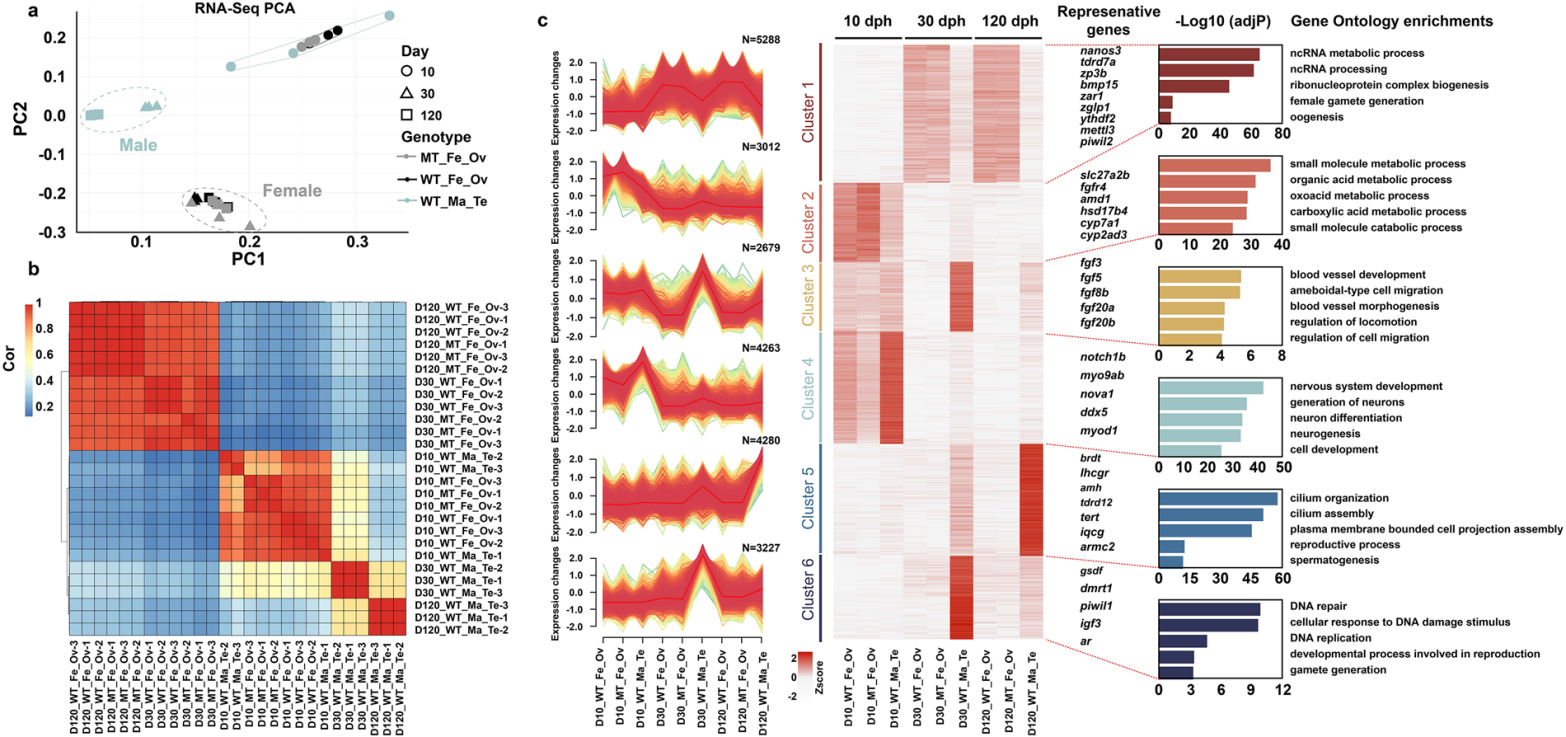
Gene expression profile based on RNA-seq among different groups. (**a**) The PCA of 27 sets of RNA-seq data. D10 is represented by circular markers, D30 by triangle markers, and D120 by square markers; MT_Fe_Ov is represented by grey, WT_Fe_Ov by black, and WT_Ma_Te by light blue. (**b**) The correlation analysis of 27 sets of RNA-seq data. (**c**) Heatmap of 6 mRNA expression profile clusters across gonad development. The bar plots on the right display selected biological process GO enrichment of relevant clusters and represent the important genes of each cluster.

The mRNA expression pattern of MT_Fe_Ov closely mirrored that of WT_Fe_Ov at each stage (D10, D30, and D120) (Fig. 3). Therefore, we grouped RNA-seq samples from both WT_Fe_Ov and MT_Fe_Ov as the Ovary group (Fe_Ov), while WT_Ma_Te samples were assigned to the Testis group (Ma_Te). Differential expression analysis was performed using DESeq2^31^ to identify DEGs between these two groups. For the Upset analysis, we established six comparison groups: (1) ovary-upregulated at D10, (2) ovary-upregulated at D30, (3) ovary-upregulated at D120, (4) testis-upregulated at D10, (5) testis-upregulated at D30, and (6) testis-upregulated at D120.

**Fig. 4:**
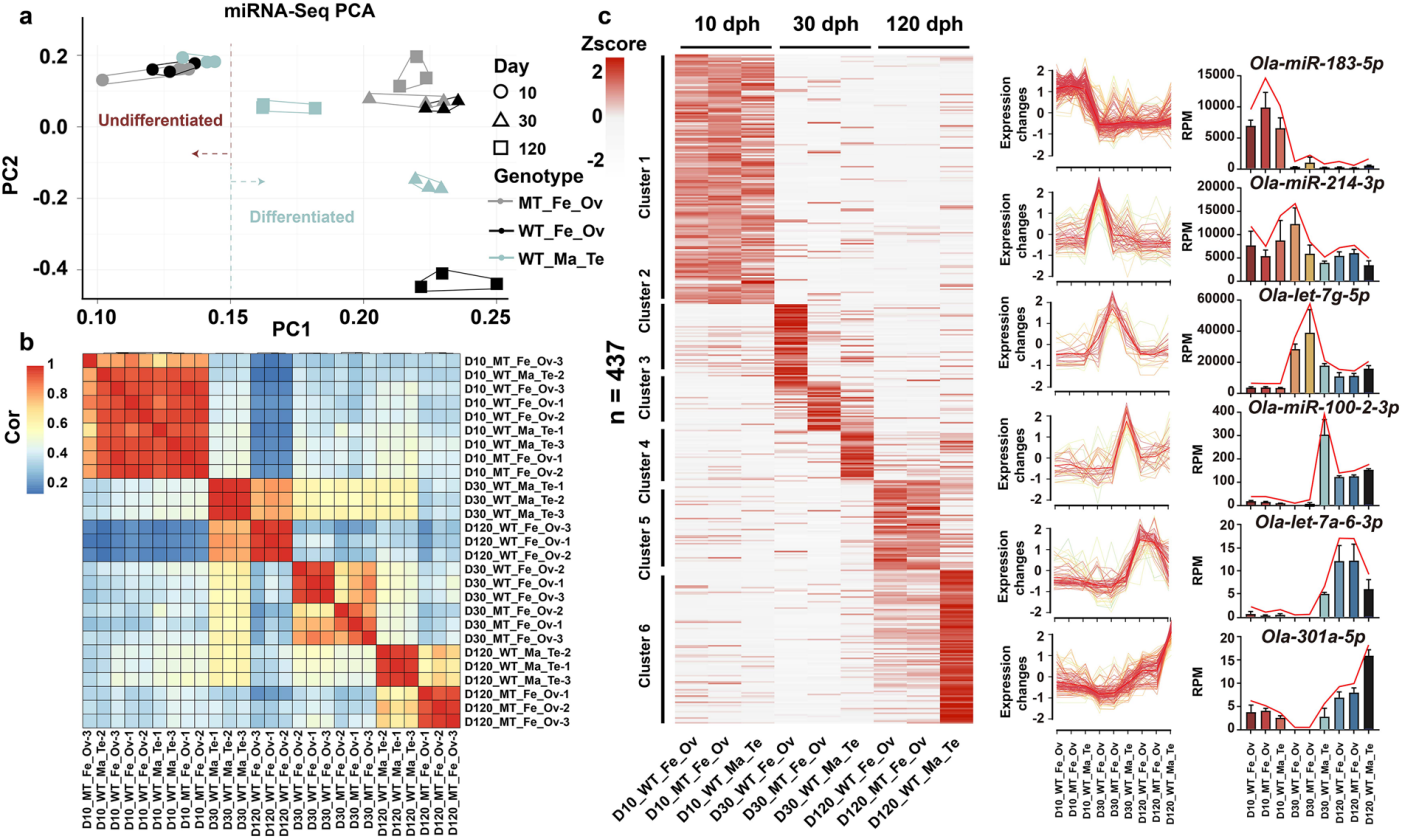
Gene expression profile based on miRNA among different groups. (**a**) PCA of 27 sets of small RNA-seq data. D10 is represented by circular markers, D30 by triangle markers, and D120 by square markers; MT_Fe_Ov is represented by grey, WT_Fe_Ov by black, and WT_Ma_Te by light blue. (**b**) Correlation analysis of 27 sets of small RNA-seq data. (**c**) Heatmap of 6 miRNA expression profile clusters across gonad development. The bar plots and line charts on the right display expressing trends of the miRNAs of each cluster.

PCA results indicated significant separation of miRNA expression patterns along PC1 for all three genotypes at both D30 and D120 (Fig. 4a). To further investigate genotype-specific effects, we expanded the Upset analysis to nine comparison groups, including pairwise contrasts between WT_Fe_Ov, MT_Fe_Ov, and WT_Ma_Te at each stage (D10, D30, and D120). This approach facilitated systematic exploration of dynamic transcriptional and post-transcriptional regulatory processes during medaka sex differentiation.

## Data Records

All raw reads from RNA-seq and small RNA-seq were deposited in the Sequence Read Archive (SRA) under the study accession SRP529327^37^. The raw read count dataset of RNA-seq is available in the NCBI Gene Expression Omnibus (GEO) under accession number GSE288571^38^, while the small RNA-seq raw read count dataset is under accession number GSE288572^39^.

We generated 27 RNA-seq libraries, producing a total of 66.23 Mb high-quality read pairs. Each library yielded between 19.8 and 36.2 million read pairs, with an average Q20 score of 94.67% (Fig. 2a, b and Table 1). Using the Medaka reference genome (ASM223467v1), we achieved an average alignment rate of 83.2% for RNA-seq reads (Fig. 2c and Table 2).

**Table 1:** Quality summary of RNA-seq data.

Sample_id	Total_reads	Total_bases	Read1 q20_rate	Read2 20_rate	Read1 q30_rate	Read2 q30_rate	GC content
D10_WT_Fe_Ov-1	23,281,142	6,984,342,600	97.37%	94.81%	92.96%	88.46%	51.25%
D10_WT_Fe_Ov-2	21,976,261	6,592,878,300	97.39%	95.16%	93.08%	89.11%	50.35%
D10_WT_Fe_Ov-3	24,072,298	7,221,689,400	97.45%	94.83%	93.20%	88.45%	51.08%
D10_MT_Fe_Ov-1	24,057,982	7,217,394,600	97.41%	94.86%	93.10%	88.59%	51.82%
D10_MT_Fe_Ov-2	24,077,566	7,223,269,800	97.26%	94.41%	92.68%	87.63%	51.48%
D10_MT_Fe_Ov-3	24,006,186	7,201,855,800	97.28%	94.44%	92.75%	87.67%	51.43%
D10_WT_Ma_Te-1	22,729,540	6,818,862,000	97.27%	94.59%	92.65%	87.89%	50.53%
D10_WT_Ma_Te-2	21,328,720	6,398,616,000	97.34%	95.38%	92.92%	89.60%	49.78%
D10_WT_Ma_Te-3	21,571,864	6,471,559,200	97.59%	95.10%	93.54%	89.04%	49.48%
D30_WT_Fe_Ov-1	24,045,970	7,213,791,000	98.21%	97.71%	93.97%	92.22%	52.63%
D30_WT_Fe_Ov-2	24,017,013	7,205,103,900	98.23%	97.75%	94.06%	92.35%	52.22%
D30_WT_Fe_Ov-3	24,131,188	7,239,356,400	98.32%	98.01%	94.42%	93.28%	52.46%
D30_MT_Fe_Ov-1	24,029,240	7,208,772,000	97.94%	96.88%	93.11%	89.82%	52.39%
D30_MT_Fe_Ov-2	24,122,503	7,236,750,900	98.06%	97.52%	93.62%	91.83%	52.34%
D30_MT_Fe_Ov-3	24,018,548	7,205,564,400	97.90%	97.32%	93.05%	91.00%	52.40%
D30_WT_Ma_Te-1	36,137,715	7,227,543,000	97.66%	95.78%	93.97%	89.72%	49.26%
D30_WT_Ma_Te-2	32,750,838	6,550,167,600	95.74%	95.67%	89.91%	89.43%	48.82%
D30_WT_Ma_Te-3	36,206,331	7,241,266,200	95.61%	95.64%	89.63%	89.33%	49.05%
D120_WT_Fe_Ov-1	24,057,418	7,217,225,400	98.11%	95.50%	94.74%	89.40%	50.98%
D120_WT_Fe_Ov-2	24,021,741	7,206,522,300	97.96%	95.24%	94.39%	88.81%	50.61%
D120_WT_Fe_Ov-3	21,406,542	6,421,962,600	98.13%	95.71%	94.86%	89.93%	51.33%
D120_MT_Fe_Ov-1	24,039,351	7,211,805,300	98.08%	95.32%	94.72%	89.01%	50.89%
D120_MT_Fe_Ov-2	24,011,544	7,203,463,200	98.07%	95.39%	94.63%	89.17%	51.03%
D120_MT_Fe_Ov-3	24,056,110	7,216,833,000	98.09%	95.51%	94.70%	89.45%	50.84%
D120_WT_Ma_Te-1	24,071,018	7,221,305,400	98.19%	96.25%	94.84%	90.51%	49.21%
D120_WT_Ma_Te-2	19,822,283	5,946,684,900	98.08%	95.17%	94.73%	88.77%	49.30%
D120_WT_Ma_Te-3	20,328,069	6,098,420,700	97.98%	95.28%	94.49%	89.03%	48.90%

D10: 10 days post hatch, D30: 30 days post hatch, D120: 120 days post hatch, WT_Ma_Te: the testis in the male medaka of the wild type, WT_Fe_Ov: the ovary in the female medaka of the wild type, and MT_Fe_Ov: the ovary in the female medaka of the mutant type, 1: sample1, 2: sample2, 3: sample3.

**Table 2:** Summary of mapping results from RNA-seq. uniq_align: reads successfully mapped to one genomic location, mul_align: reads successfully mapped to multiple genomic locations, overall_align: overall alignment.

Sample_id	Paired_reads	Uniq_align	Mul_align	%Uniq align	%Mul align	%Overall align
D10_WT_Fe_Ov-1	23,281,142	18,725,367	657,099	80.43	2.82	83.25
D10_WT_Fe_Ov-2	21,976,261	18,081,832	530,588	82.28	2.41	84.69
D10_WT_Fe_Ov-3	24,072,298	19,494,700	649,970	80.98	2.7	83.68
D10_MT_Fe_Ov-1	24,057,982	19,921,514	601,808	82.81	2.5	85.31
D10_MT_Fe_Ov-2	24,077,566	19,950,003	606,688	82.86	2.52	85.38
D10_MT_Fe_Ov-3	24,006,186	19,487,427	568,900	81.18	2.37	83.55
D10_WT_Ma_Te-1	22,729,540	18,811,141	417,375	82.76	1.84	84.6
D10_WT_Ma_Te-2	21,328,720	17,456,543	456,862	81.85	2.14	83.99
D10_WT_Ma_Te-3	21,571,864	17,214,423	471,822	79.8	2.19	81.99
D30_WT_Fe_Ov-1	24,045,970	19,080,585	1,949,950	79.35	8.11	87.46
D30_WT_Fe_Ov-2	24,017,013	19,191,494	1,963,067	79.91	8.17	88.08
D30_WT_Fe_Ov-3	24,131,188	19,207,646	1,989,848	79.6	8.25	87.84
D30_MT_Fe_Ov-1	24,029,240	17,489,461	1,685,097	72.78	7.01	79.8
D30_MT_Fe_Ov-2	24,122,503	17,101,675	1,847,192	70.9	7.66	78.55
D30_MT_Fe_Ov-3	24,018,548	18,283,159	1,560,893	76.12	6.5	82.62
D30_WT_Ma_Te-1	36,137,715	25,727,009	1,306,939	71.19	3.62	74.81
D30_WT_Ma_Te-2	32,750,838	22,895,786	1,142,649	69.91	3.49	73.4
D30_WT_Ma_Te-3	36,206,331	24,919,107	1,285,803	68.83	3.55	72.38
D120_WT_Fe_Ov-1	24,057,418	17,968,462	2,274,139	74.69	9.45	84.14
D120_WT_Fe_Ov-2	24,021,741	18,112,448	2,194,695	75.4	9.14	84.54
D120_WT_Fe_Ov-3	21,406,542	16,222,158	1,846,463	75.78	8.63	84.41
D120_MT_Fe_Ov-1	24,039,351	18,201,029	2,149,305	75.71	8.94	84.65
D120_MT_Fe_Ov-2	24,011,544	18,159,465	2,242,203	75.63	9.34	84.97
D120_MT_Fe_Ov-3	24,056,110	18,104,040	2,299,542	75.26	9.56	84.82
D120_WT_Ma_Te-1	24,071,018	20,608,761	729,351	85.62	3.03	88.65
D120_WT_Ma_Te-2	19,822,283	16,289,666	587,134	82.18	2.96	85.14
D120_WT_Ma_Te-3	20,328,069	16,843,364	603,727	82.86	2.97	85.83

In parallel, we constructed 27 small RNA-seq libraries, generating 66.19 Mb clean read pairs, with individual libraries ranging from 20.3 to 30.3 million read pairs. Over 98% of small RNA-seq reads exhibited a quality score of ≥Q30 (Fig. 2d,e and Table 3). Alignment analysis revealed that 40.55% of small RNA-seq reads mapped to the Medaka genome. To identify known miRNAs, we aligned reads to the FishmiRNA database (https://www.fishmirna.org), achieving alignment rates ranging from 0.2% to 36%, with an average of 11% (Table 4).

**Table 3:** Quality summary of small RNA-seq data.

Sample_id	Total_reads	Total_bases	Q30_rate	Mean_length	GC_content
D10_WT_Fe_Ov-1	25,340,740	610,953,125	98.61%	24.11	54.51%
D10_WT_Fe_Ov-2	25,294,606	724,311,270	98.43%	28.64	57.20%
D10_WT_Fe_Ov-3	25,403,760	688,345,355	98.72%	27.1	56.78%
D10_MT_Fe_Ov-1	25,233,553	628,155,052	98.79%	24.89	55.04%
D10_MT_Fe_Ov-2	25,211,933	678,457,284	98.74%	26.91	58.92%
D10_MT_Fe_Ov-3	25,197,125	600,346,800	98.76%	23.83	53.18%
D10_WT_Ma_Te-1	25,335,072	741,052,665	98.78%	29.25	60.01%
D10_WT_Ma_Te-2	25,343,246	748,628,615	98.58%	29.54	59.37%
D10_WT_Ma_Te-3	25,255,462	674,551,143	98.81%	26.71	54.39%
D30_WT_Fe_Ov-1	20,338,240	531,257,027	98.05%	26.12	47.55%
D30_WT_Fe_Ov-2	20,409,172	549,499,676	97.92%	26.92	46.62%
D30_WT_Fe_Ov-3	20,381,851	544,816,277	98.40%	26.73	47.40%
D30_MT_Fe_Ov-1	20,423,310	550,871,375	98.31%	26.97	47.34%
D30_MT_Fe_Ov-2	20,437,592	554,195,057	98.21%	27.12	46.36%
D30_MT_Fe_Ov-3	20,463,766	563,557,583	98.16%	27.54	45.66%
D30_WT_Ma_Te-1	20,430,027	536,221,818	98.21%	26.25	46.28%
D30_WT_Ma_Te-2	20,443,465	521,292,557	98.24%	25.5	46.64%
D30_WT_Ma_Te-3	20,522,479	542,763,725	98.15%	26.45	46.25%
D120_WT_Fe_Ov-1	25,378,734	762,354,361	98.91%	30.04	49.16%
D120_WT_Fe_Ov-2	27,838,712	795,911,644	99.06%	28.59	46.43%
D120_WT_Fe_Ov-3	27,809,439	802,474,248	99.08%	28.86	46.45%
D120_MT_Fe_Ov-1	25,318,727	736,735,418	98.91%	29.1	48.18%
D120_MT_Fe_Ov-2	27,835,139	804,221,984	98.78%	28.89	46.76%
D120_MT_Fe_Ov-3	25,347,716	733,508,948	98.69%	28.94	48.43%
D120_WT_Ma_Te-1	30,352,896	830,596,724	99.32%	27.36	49.93%
D120_WT_Ma_Te-2	30,323,352	853,869,484	99.15%	28.16	50.07%
D120_WT_Ma_Te-3	30,317,565	841,580,257	99.45%	27.76	50.32%

**Table 4:** Summary of mapping results from small RNA-seq.

Sample_id	Mapping to genome			Mapping to miRNA		
	Total reads	Mapped reads	%Mapped reads	Total reads	Mapped reads	%Mapped reads
D10_WT_Fe_Ov-1	25,246,839	12,306,815	48.746	25,245,658	7,316,420	28.98%
D10_WT_Fe_Ov-2	25,190,914	9,453,796	37.529	25,189,076	4,245,665	16.86%
D10_WT_Fe_Ov-3	25,355,774	12,545,104	49.476	25,354,463	4,747,317	18.72%
D10_MT_Fe_Ov-1	25,117,208	12,646,029	50.348	25,114,756	7,141,964	28.44%
D10_MT_Fe_Ov-2	25,043,549	13,333,089	53.24	25,041,417	5,245,343	20.95%
D10_MT_Fe_Ov-3	24,260,188	12,792,958	52.732	24,259,427	8,683,119	35.79%
D10_WT_Ma_Te-1	24,312,272	8,823,057	36.291	24,310,479	2,487,793	10.23%
D10_WT_Ma_Te-2	25,230,729	9,295,207	36.841	25,228,181	2,481,530	9.84%
D10_WT_Ma_Te-3	25,077,244	13,506,466	53.859	25,075,839	8,654,194	34.51%
D30_WT_Fe_Ov-1	29,252,111	14,036,728	47.985	28,616,209	803,915	2.81%
D30_WT_Fe_Ov-2	29,775,099	14,211,937	47.731	29,181,006	527,847	1.81%
D30_WT_Fe_Ov-3	29,546,350	13,925,452	47.131	28,853,610	610,739	2.12%
D30_MT_Fe_Ov-1	25,311,035	10,255,265	40.517	25,283,070	109,731	0.43%
D30_MT_Fe_Ov-2	22,897,912	6,897,848	30.124	22,868,748	55,913	0.24%
D30_MT_Fe_Ov-3	25,087,756	9,841,288	39.227	25,058,031	333,267	1.33%
D30_WT_Ma_Te-1	27,391,880	10,748,511	39.24	27,378,402	534,595	1.95%
D30_WT_Ma_Te-2	27,822,632	11,532,499	41.45	27,810,030	490,731	1.76%
D30_WT_Ma_Te-3	27,809,439	11,257,716	40.482	27,796,786	305,367	1.10%
D120_WT_Fe_Ov-1	19,854,811	6,419,342	32.331	19,790,498	1,822,442	9.21%
D120_WT_Fe_Ov-2	19,485,178	6,771,627	34.753	19,422,167	3,478,664	17.91%
D120_WT_Fe_Ov-3	19,839,870	6,406,025	32.289	19,776,203	2,026,439	10.25%
D120_MT_Fe_Ov-1	19,846,791	6,514,453	32.824	19,784,053	1,615,155	8.16%
D120_MT_Fe_Ov-2	19,558,876	6,700,977	34.261	19,496,366	2,599,139	13.33%
D120_MT_Fe_Ov-3	20,085,501	6,056,564	30.154	20,023,192	1,378,535	6.88%
D120_WT_Ma_Te-1	19,709,852	6,901,131	35.014	19,646,638	1,142,285	5.81%
D120_WT_Ma_Te-2	19,748,525	6,917,745	35.029	19,684,427	1,045,487	5.31%
D120_WT_Ma_Te-3	20,013,972	6,893,649	34.444	19,952,636	1,137,579	5.70%

## Technical Validation

### mRNA expression analysis

RNA-seq analysis was performed with three biological replicates per group. Sample consistency was confirmed by correlation coefficients exceeding 0.8 within each group (Fig. 3a, b). Using FPKM values, principal component analysis (PCA) and correlation analysis were conducted. PCA revealed clear separation of testis and ovary samples along principal component 1 (PC1, x-axis), further supported by correlation analysis (Fig. 3a, b). Based on expression patterns, mRNAs were clustered into six groups, representing distinct expression trends in MT_Fe_Ov, WT_Fe_Ov, and WT_Ma_Te across gonadal development stages (Fig. 3c). To validate the RNA-seq results, we performed qPCR on genes from each cluster (Figure S1 and Table S1). Differential expression analysis of mRNA identified significant changes between groups at each stage, demonstrating proper experimental design, high-quality data, and good technical reproducibility (Table 5 and Figure S2).

**Table 5:** Summary of DEGs across experimental groups. DEGs were defined as genes with |log2FoldChange| ≥ 1 and an adjusted *P*-value ≤ 0.05.

Stage	Group1	Group2	Up_DEGs	Down_DEGs	Sum_DEGs
D10	WT_Fe_Ov	WT_Ma_Te	133	14	147
	MT_Fe_Ov	WT_Ma_Te	588	356	944
	WT_Fe_Ov	MT_Fe_Ov	137	223	360
D30	WT_Fe_Ov	WT_Ma_Te	5,244	7,245	12,489
	MT_Fe_Ov	WT_Ma_Te	4,300	7,253	11,553
	WT_Fe_Ov	MT_Fe_Ov	328	296	624
D120	WT_Fe_Ov	WT_Ma_Te	5,193	5,965	11,158
	MT_Fe_Ov	WT_Ma_Te	5,195	6,155	11,350
	WT_Fe_Ov	MT_Fe_Ov	42	56	98

### miRNA expression analysis

Small RNA-seq analysis was performed with three biological replicates per group. Sample consistency was confirmed by correlation coefficients exceeding 0.8 within each group (Fig. 4a, b). Using FPKM values, PCA and correlation analysis were conducted. PCA indicated that D10 samples from all genotypes clustered together, while D30 and D120 samples formed distinct clusters, a pattern corroborated by correlation analysis (Fig. 4a, b). miRNA expression patterns were divided into six clusters, representing distinct trends in MT_Fe_Ov, WT_Fe_Ov, and WT_Ma_Te across gonadal development stages (Fig. 4c). To ensure the quality of small RNA-seq data, we performed qPCR on miRNAs from each cluster (Figure S3 and Table S1). These results demonstrate the conservation and reusability of the miRNA data. Differential expression analysis of miRNAs revealed significant differences between MT_Fe_Ov, WT_Fe_Ov, and WT_Ma_Te across all stages, validating the biological plausibility of the experimental data (Table 6 and Figure S4).

**Table 6:** Summary of DEMs across experimental groups. DEMs were defined as miRNAs with |log2FoldChange| ≥ 1 and an adjusted *P*-value ≤ 0.05.

Stage	Group1	Group2	Up_DEMs	Down_DEMs	Sum_DEMs
D10	WT_Fe_Ov	WT_Ma_Te	13	4	17
	MT_Fe_Ov	WT_Ma_Te	10	0	10
	WT_Fe_Ov	MT_Fe_Ov	0	0	0
D30	WT_Fe_Ov	WT_Ma_Te	49	107	156
	MT_Fe_Ov	WT_Ma_Te	43	74	117
	WT_Fe_Ov	MT_Fe_Ov	23	34	57
D120	WT_Fe_Ov	WT_Ma_Te	72	59	131
	MT_Fe_Ov	WT_Ma_Te	60	70	130
	WT_Fe_Ov	MT_Fe_Ov	1	1	2

### Time-series analysis of DEGs and DEMs

**Fig. 5:**
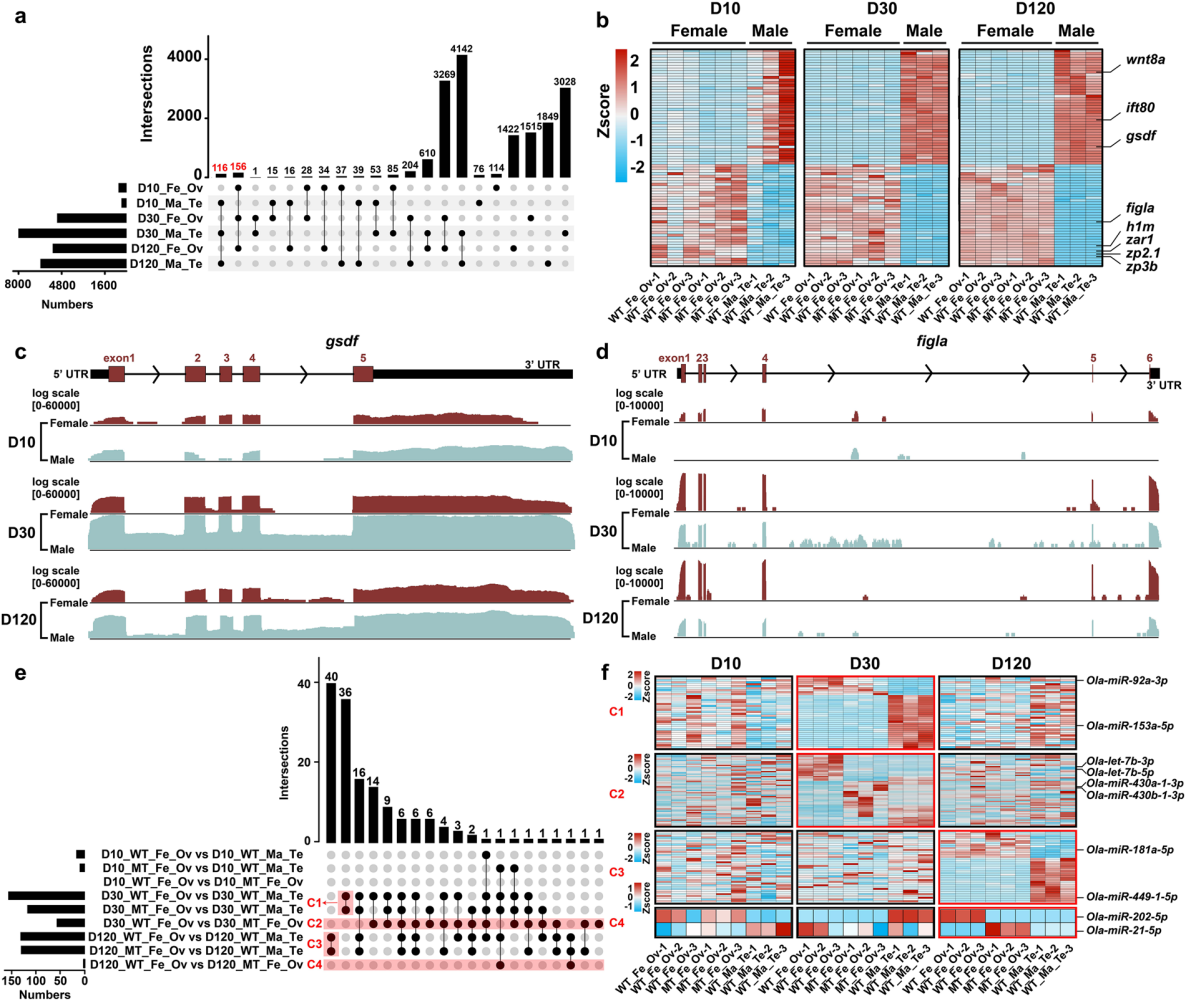
Transcriptional and post-transcriptional dynamic analysis of gonadal development. (**a**) UpSet plots indicate numbers of the common and specific DEGs among different groups. D10_Fe_Ov: Ovary of female medaka at D10; D10_Ma_Te: Testis of male medaka at D10; D30_Fe_Ov: Ovary of female medaka at D30; D30_Ma_Te: Testis of male medaka at D30; D120_Fe_Ov: Ovary of female medaka at D120; D120_Ma_Te: Testis of male medaka at D120. (**b**) The heatmap represents the expression level of DEGs are common in the testis and ovary at the D10, D30, and D120 development phases. (**c**) The expression pattern of the *gsdf* in the gonad of medaka. The red boxes represent exon regions, with exon numbers indicated by digits. The black boxes denote UTR regions, and the black arrows indicate the transcription direction. (**d**) The expression pattern of the *figlα* in the gonad of medaka. The red boxes represent exon regions, with exon numbers indicated by digits. The black boxes denote UTR regions, and the black arrows indicate the transcription direction. (**e**) UpSet plots indicate numbers of the common and specific DEMs among different groups. C1 represents the common DEMs between WT_Fe_Ov and MT_Fe_Ov at D30 compared to WT_Ma_Te. C2 represents the DEMs between WT_Fe_Ov and MT_Fe_Ov at D30. C3 represents the common DEMs between WT_Fe_Ov and MT_Fe_Ov at D120 compared to WT_Ma_Te. C4 represents the DEMs between WT_Fe_Ov and MT_Fe_Ov at D120. (**f**) The heatmap illustrates the expression levels of DEMs in C1, C2, C3, and C4.

Comparative analysis revealed 116 testis-associated DEGs (e.g., *gsdf*) with persistently high expression across three stages (Fig. 5a – c), contrasting with 156 ovary-enriched DEGs (e.g., *figlα*) showing progressive upregulation (Fig. 5d). miRNA screening identified 40 and 21 DEMs in the between the Fe_Ov and Ma_Te groups at D30 and D120, respectively. Additionally, 57 and 2 DEMs were detected between the WT_Fe_Ov and MT_Fe_Ov groups at the corresponding time points (Fig. 5e,f). These results provided a foundational regulatory atlas for gonadal development in medaka.

### Prediction of medaka miRNA targets

To systematically map miRNA-mRNA interactions, we developed a novel computational workflow using EasyTargetScan_0.2.py for prediction. Utilizing the 437 miRNAs and 18,205 3′UTR sequences in medaka, we identified 807,827 miRNA-mRNA interaction pairs with prediction scores (Table S2). This robust approach addresses concerns about evolutionary divergence while maintaining TargetScan’s rigorous computational framework. Our study represents the application of TargetScan for miRNA research in medaka, offering a valuable resource for the scientific community.

## Supplementary information

Supplementary Information

Table S1

Table S2

## Data Availability

The Methods sections clearly describe the parameters used in this study, all of which are publicly available. If the developer did not specify detailed parameters for a particular software, we used the default parameters as suggested. The processed files and code for the analysis pipelines was deposited at Zenodo: 10.5281/zenodo.15208230^36^.
