# Supplementary material for: Integrating gonadal RNA-seq and small RNA-seq to analyze mRNA and miRNA changes in medaka sex differentiation
Source: Sci Data. 2025 May 12;12:778. doi: 10.1038/s41597-025-05129-y (PMC12069655; doi:10.1038/s41597-025-05129-y)
Supplement: Supplementary file 1 — Supplementary Information [file 41597_2025_5129_MOESM1_ESM.docx]

**Contents**

Supplementary Figure 1 -----------------------------------------------------------------------1

Supplementary Figure 2 -----------------------------------------------------------------------2

Supplementary Figure 3 -----------------------------------------------------------------------3

Supplementary Figure 4 -----------------------------------------------------------------------4

**Supplementary Figure 1: Validation of RNA-Seq data by qPCR.**


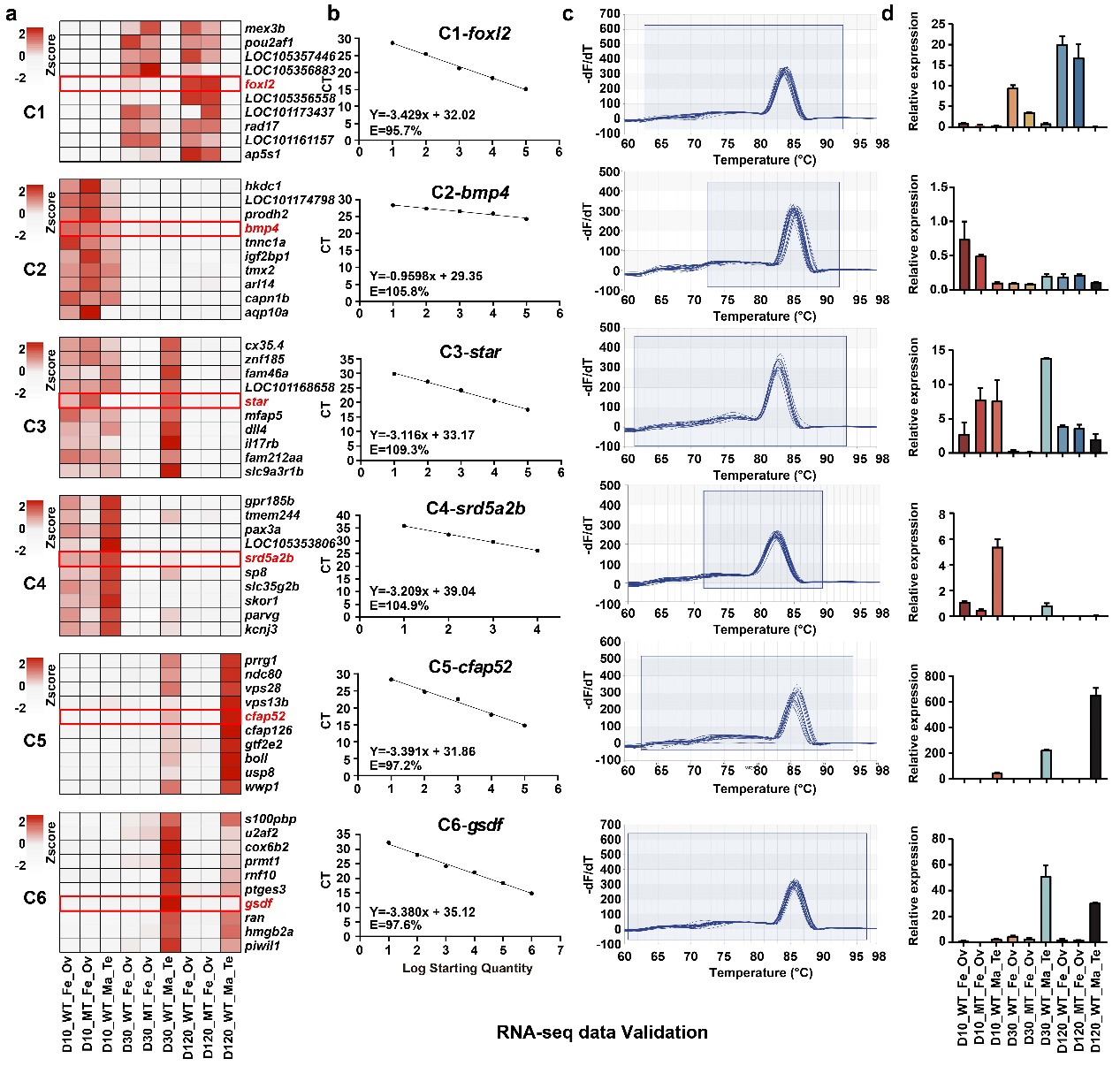


a: Heatmaps displaying the FPKM expression patterns of genes from each cluster in RNA-seq data, with validated genes marked by red box. b: Amplification efficiency of qPCR primers for validated genes. c: Specificity of qPCR primers for validated genes. d: Bar plots showing qPCR validation results of genes from each cluster.

1

**Supplementary Figure 2: Volcano plot of DEGs analysis among different groups.**


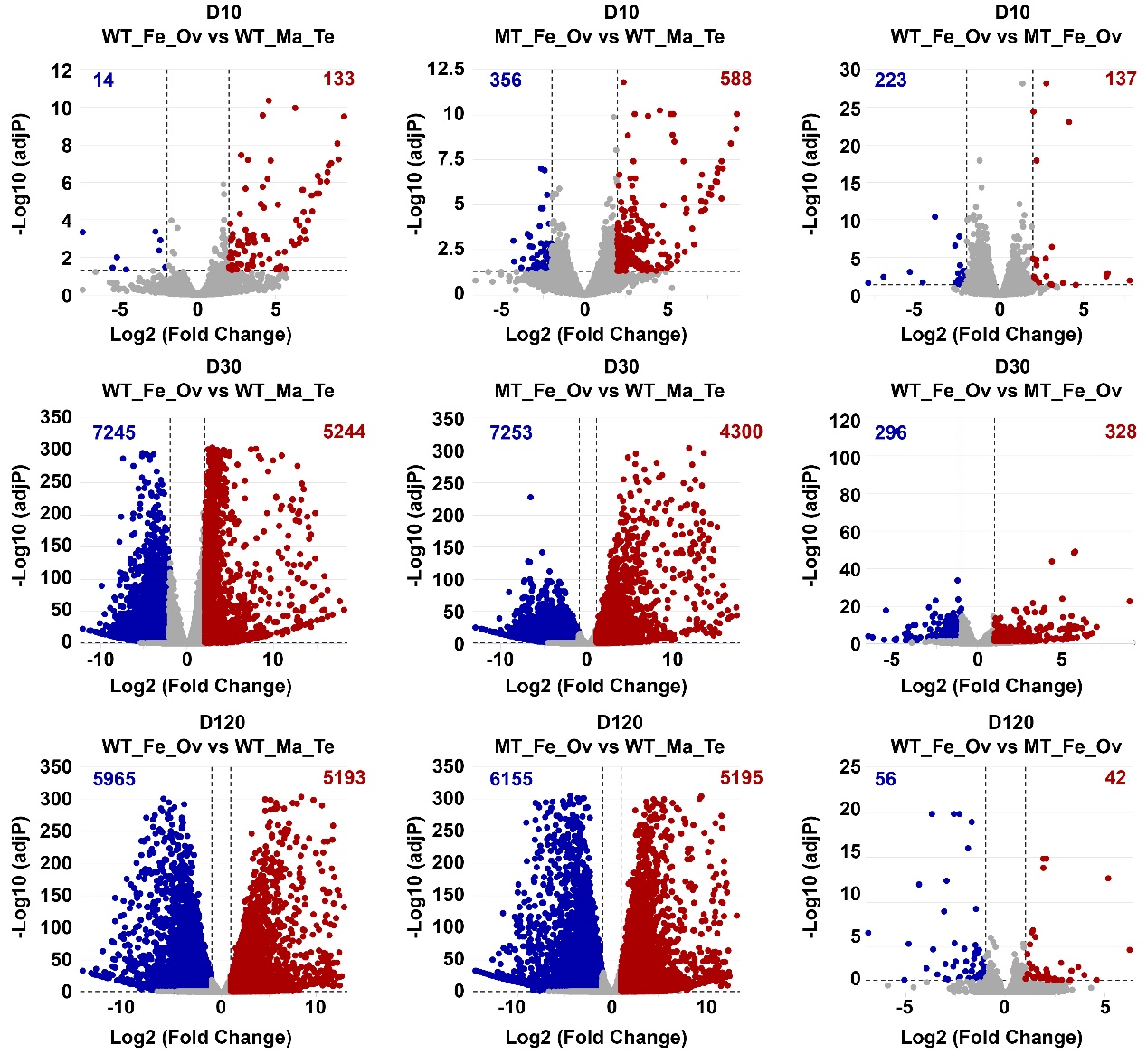


2

Each dot represents each gene, and red dots stand for up-regulated DEGs; blue dots stand for down-regulated DEGs.

a: Comprehensive analysis of the number of isoforms per gene for short-read assembled, long-read assembled, NCBI, and Ensembl isoforms.

b: Comprehensive analysis of the number of exons per isoform for short-read assembled, long-read assembled, NCBI, and Ensembl isoforms.

c: Proportion of SQANTI structural categories for short-read assembled isoforms and long-read assembled isoforms compared with the NCBI and Ensembl annotations. FSM: matches all splice junction (SJs) perfectly; ISM: matches the reference SJs partially; NIC: novel isoform with a new combination of known splice sites; NNC: novel isoforms with at least a new splicing site; Genic: within an intron or overlaps introns and exons; Antisense: in the reverse strand to the reference isoform; Fusion: overlap with isoforms from more than 1 gene; Intergenic: do not overlap with any isoform.

d: Schematic diagrams of different classes of transcripts generated from SQANTI3.

**Supplementary Figure 3: Validation of small RNA-Seq data by qPCR.**


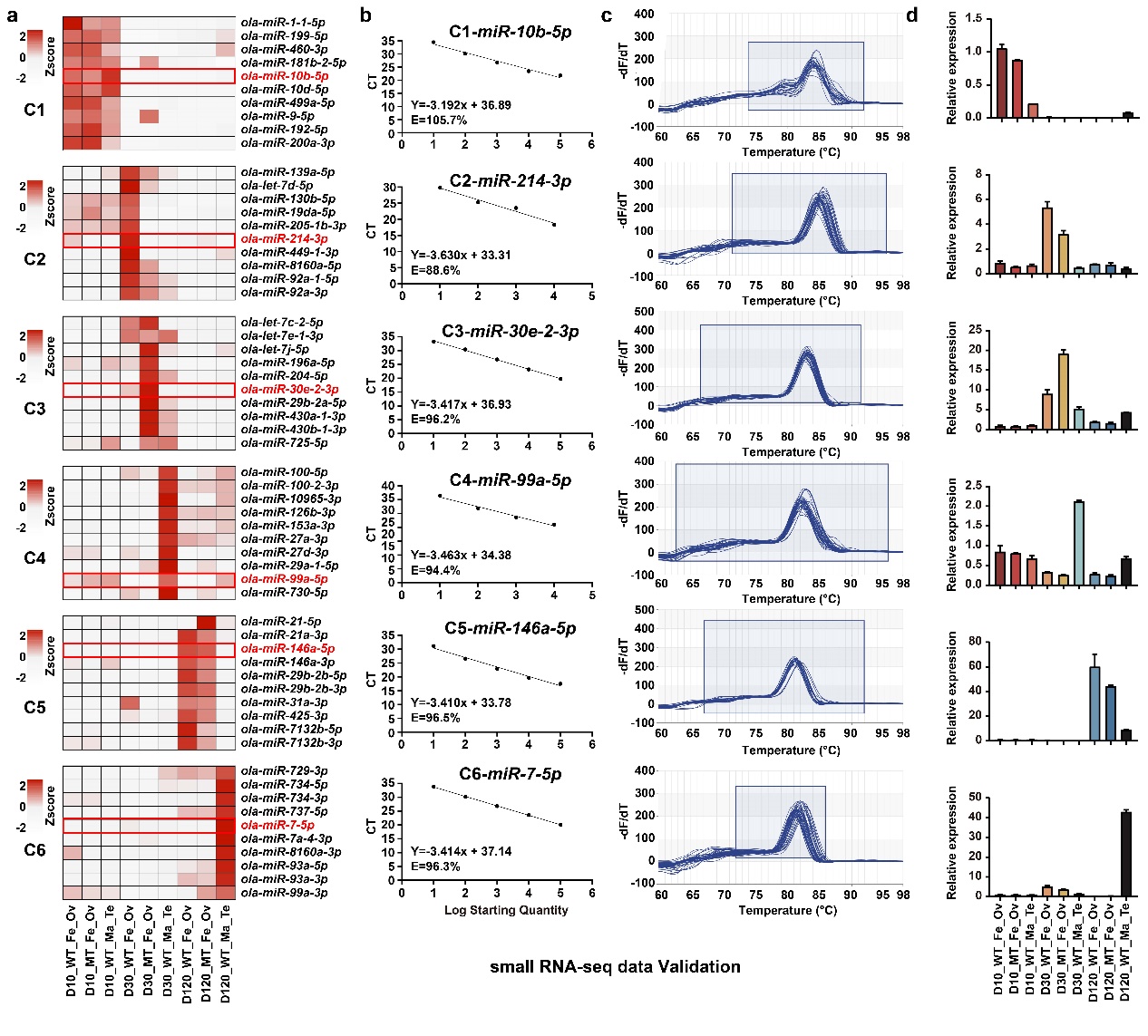


a: Heatmaps displaying the RPM expression patterns of miRNAs from each cluster in miRNA-seq data, with validated miRNAs marked by red box. b: Amplification efficiency of qPCR primers for each validated miRNA. c: Specificity of qPCR primers for each validated miRNA. d: Bar plots showing qPCR validation results of miRNAs from each cluster.

3

**Supplementary Figure 4: Volcano plot of DEMs analysis among different groups.**


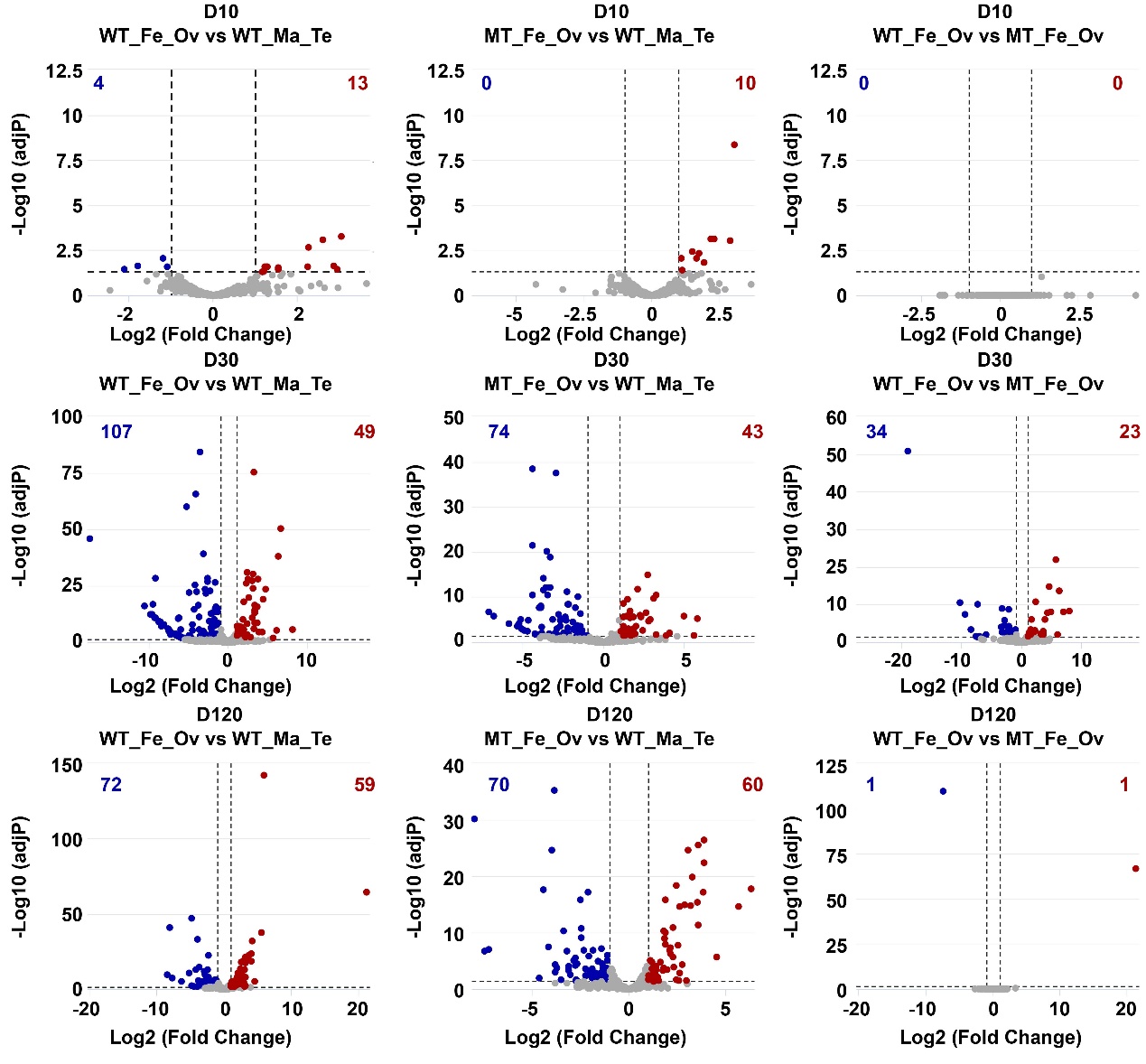


4

Each dot represents each gene, and red dots stand for up-regulated DEMs; blue dots stand for down-regulated DEMs.
